# Depression, anxiety, and poor well-being at discharge from psychiatric hospitals: prevalence and risk factors

**DOI:** 10.3389/fpsyt.2024.1408095

**Published:** 2024-07-11

**Authors:** Wanying Mao, Reham Shalaby, Ernest Owusu, Hossam Eldin Elgendy, Belinda Agyapong, Ejemai Eboreime, Peter Silverstone, Pierre Chue, Xin-Min Li, Wesley Vuong, Arto Ohinmaa, Valerie Taylor, Andrew J. Greenshaw, Vincent I. O. Agyapong

**Affiliations:** ^1^ Department of Psychiatry, University of Alberta, Edmonton, AB, Canada; ^2^ Department of Psychiatry, Dalhousie University, Halifax, NS, Canada; ^3^ Alberta Health Services, Addiction and Mental Health Services, Edmonton, AB, Canada; ^4^ School of Public Health, University of Alberta, Edmonton, AB, Canada; ^5^ Department of Psychiatry, Cumming School of Medicine, University of Calgary, Calgary, AB, Canada

**Keywords:** depression, anxiety, well-being, mental health, psychiatric hospital discharge

## Abstract

**Background:**

Being ready for discharge is vital to successful hospital-to-home transitions. For many patients, however, the transition from psychiatric hospital care to outpatient care can be challenging. An in-depth understanding of the mental health conditions of patients at discharge is crucial and instructive for recovery research.

**Objective:**

The purpose of this study was to determine the prevalence and risk factors of depression, anxiety, and poor well-being symptoms among patients who are about to be discharged from psychiatric units in Alberta, Canada. Our aim was to help determine the prevalence of anxiety, depression, and overall well-being among the general psychiatric inpatient population in Alberta before discharge and the potential factors which may influence this.

**Methods:**

This epidemiological study used a cross-sectional quantitative survey from March 8, 2022, to November 5, 2023, to assess depression, anxiety, and well-being. Participants were invited to complete an online questionnaire that contained demographics, clinical information, and responses to the PHQ-9, GAD-7, and WHO-5 questionnaires. SPSS version 25 was used to analyze the data. Descriptive, univariate, and multivariate regression analyses were employed.

**Result:**

The study found that the prevalence of likely depression, anxiety, and poor well-being among patients about to be discharged was 37.1%, 56.4%, and 48.3%, respectively. Based on a logistic regression model, there was a statistically significant association between anxiety, depression, and poor well-being diagnoses and multiple socio-demographic and clinical factors such as ethnicity, primary mental health diagnoses, education level, housing status, depression, anxiety, and well-being at baseline.

**Conclusion:**

Mental health assessment at discharge is a critical step in the recovery and transition of care. There is still a need for further research to identify the underlying causes and robust predictors of mental health symptoms in patients about to be discharged and to provide appropriate interventions and supportive resources both before and following discharge. Future research utilizing these findings may help identify key opportunities to improve outcomes for patients after discharge.

## 1 Introduction

Mental illness causes profound health, social, cultural, and economic problems all over the world. In most cases, mental disorders are chronic and complex, characterized by frequent occurrences of acute symptoms (1). There was considerable attention paid to hospital discharge and recovery of mental health problems for decades. Even so, psychiatric hospitalizations and readmissions remain high in many countries (1–3). The study of hospital discharge among populations with various mental health conditions and the identification of effective strategies and interventions for promoting recovery are currently in their infancy (1). There has been much discussion regarding empowerment, hope, responsibility, peer support, advocacy, and quality of life in the recovery debate. Notwithstanding, these concepts remain unclear without a thorough understanding of patients’ mental health conditions at discharge (1, 4).

Being ready for discharge is important to improve successful hospital-to-home transitions. However, for many patients, the transition from in-patient psychiatric hospital treatment to outpatient care can prove difficult. The uncertainty of the future may leave individuals feeling frustrated and helpless as they face numerous challenges ahead (3, 5). Research has shown that although the transition from hospital to community is usually associated with positive perceptions of recovery, it is also often associated with negative perceptions of a lack of health monitoring and support after discharge (6). The presence of these factors may exacerbate symptoms of anxiety and depression associated with “relocation stress.” According to Carpenito-Moyet “relocation stress” is defined as: “a state in which a person experiences physiologic or psychological disturbances as a result of transfer from one environment to another” (7). It has been observed that elevated levels of anxiety and depression symptoms, as well as poor well-being, are significant problems among several patients at discharge; aside from the increased risk of readmission and ED visits, they are associated with serious, potentially life-threatening adverse outcomes, such as homelessness (5, 8), violent behavior (9, 10), and even involvement in criminal activities (3, 5, 9).

During the past few decades, a growing body of literature has been published on the mental health status of patients at discharge and its influencing factors (11). It has been noted that a significant proportion of patients were not ready to be discharged from hospitals. Factors influencing patients’ readiness to be discharged from hospital include age, marital status, hospitalization length, depression, anxiety, and the quality of discharge education (11–15). However, most evidence came from patients with somatic diseases (11). Among patients with mental health disorders, however, a dearth of literature was available. There were a couple of studies that were conducted among patients with schizophrenia and anorexia nervosa (16, 17). In addition, two other studies focused on patients suffering from depression in China, however, without considering influencing factors (11, 18). In terms of mental health conditions at discharge, there are several research studies examining anxiety (19, 20); however, little information is available on depression and well-being.

It is widely recognized that depression and anxiety are highly comorbid (21–23). According to the Anxiety and Depression Association of America, nearly one-half of those diagnosed with depression also suffer from anxiety disorders, and converse applies (22). Furthermore, research evidence has demonstrated that levels of anxiety and depression are inversely related to levels of well-being (24). Consequently, studies focusing on depression, anxiety, and well-being status at the time of discharge should have a significant academic and practical impact.

Identifying the factors that promote or hinder the recovery process of people living with serious mental illness remains one of the most critical aspects of mental health care. Considering the background, the goal of this paper was to examine the prevalence and the sociodemographic and clinical correlates of likely depression, likely anxiety, and poor well-being among patients ready for discharge from inpatient psychiatric units in 11 acute care hospitals across the province of Alberta, Canada. As few studies have been conducted on the prevalence and risk factors of depression, anxiety, and well-being among patients with all kinds of mental health diagnoses at the time of hospital discharge from psychiatric units, it is anticipated that our research will provide insight into the most relevant factors for a successful transition into the community. To help better design future studies we aimed to collect evidence regarding clinical factors such as primary diagnosis, sociodemographic factors such as age, sex and gender, and could then utilize these findings to help determine the most appropriate interventions to improve symptoms of anxiety, depression, and overall well-being among the study participants. Standardized tools to measure scores for these symptoms would help allow identification of potential future areas for intervention. The goal from this study is thus to provide evidence and baseline data for future research in this critical area.

## 2 Methodology

### 2.1 Study setting and design

This study was conducted in Alberta, Canada, which has a population of 4,695,290. The data in this study was collected as baseline data of an original study that used a pragmatic stepped-wedge cluster-randomized, longitudinal approach to provide supportive text messages (Text4Support) and peer support services (PSS) across ten acute hospital sites in three of Alberta’s five health zones. The original study was launched in March 2022 with the objective of improving the care and support provided to patients discharged from inpatient psychiatry units and referred to community mental health services. Detailed information about this study can be found in the study protocol (2). In the present epidemiological study, patients who were ready to be discharged to the community were recruited and asked to complete a baseline survey from general adult acute care units of Alberta hospitals. According to our health utilization data, patients’ length of stay ranged from 2 to 238 days, with an average of 28.3 days and a median of 21 days. Discharge decisions are made at inpatient multidisciplinary team (MDT) meetings which are attended by the most responsible psychiatrists, nursing staff, social workers, psychologists, and occupational therapists. Decisions regarding discharge readiness are made by the MDT by consensus based on patients’ responses to treatment and positive changes in mental status. The data in the present study did not use randomization or interventions as it only aimed to examine the prevalence and correlates of mental health symptoms among patients who were ready to be discharged from hospital.

### 2.2 Data collection and inclusion criteria

On March 8, 2022, the study research team began recruiting participants across ten main sites across Edmonton, Calgary, and Grand Prairie in Alberta through a face-to-face interview. Psychiatric patients who were about to be discharged from psychiatry units in the designated hospitals were identified by the nurse manager to the research assistant. Detailed information about this project was provided to all eligible patients in a leaflet. Patients who were interested in participating signed a paper-based consent form and were invited to complete a baseline survey on a tablet through a self-administered online questionnaire. The survey was designed on REDCap (25), a secure browser-based application for building and managing online surveys and translational research databases with assistance from research team members. Essential sociodemographic factors (e.g., age, sex, ethnicity, relationship status, etc.) and clinical factors (e.g., diagnosis and levels of anxiety, depression, and well-being) were collected. For outcomes reported in this study, the study recruitment process continued until November 5, 2023, and all participants were included in the analysis.

To be eligible for the original study of which this is a sub-study, participants had to be diagnosed with any mental health illness, were about to be discharged from an inpatient psychiatry unit, be aged 18 and above, and to own a mobile device with an active phone number. Participants also needed to be able to receive text messages, read English texts, and to provide written informed consent. Patients were excluded from this study if they intended to travel out of town during the 6-month follow-up period. Phone numbers and healthcare numbers were collected and considered primary identifiers of the study participants.

### 2.3 Ethics statement

Ethical approval for this study was obtained from the University of Alberta’s Health Research Ethics Board (Ref # Pro00111459). The regional health authority also provided additional operational approval. Written informed consent was obtained from all participants.

### 2.4 Outcome measures

Prevalence and predictors of likely Generalized Anxiety Disorder (GAD), likely Major Depressive Disorder (MDD) and poor well-being were the main outcome measures of the study. The Generalized Anxiety Disorder-7 (GAD-7) questionnaire, the Patient Health Questionnaire-9 (PHQ-9) and the World Health Organisation-Five Well-Being Index (WHO-5) were used to measure each outcome, respectively.

#### 2.4.1 Generalized anxiety disorder (GAD-7)

The GAD-7 questionnaire was used to assess anxiety symptoms among respondents. The severity of GAD-related symptoms over the past two weeks is assessed using seven self-report items. The responses to each question were: Not at all sure (0), Several days (1), Over half the days (2), and nearly every day (3). There was a range of scores from 0 to 21, with higher scores indicating more severe symptoms of GAD, and a score of ten or higher is recommended as a cut-off for identifying patients with likely GAD (26). Research and clinical practice have recommended the GAD-7 scale as the most valid tool to assess the severity of GAD symptoms (26, 27). With a threshold score of 10, the GAD-7 has a sensitivity of 89% and a specificity of 82% for generalized anxiety disorder. Additionally, the GAD-7 demonstrated good test-retest reliability (intraclass correlation = 0.83), and excellent internal consistency (Cronbach α = 0.92) (26).

#### 2.4.2 Patient Health Questionnaire-9 (PHQ-9)

The PHQ-9 is a self-report measure based on the 9 DSM-IV criteria for major depression. Patients are asked about symptoms they experienced two weeks before answering questionnaires. Scores range from never (0), several days (1), more than half of days (2) to 3 (nearly every day), with every item ranging from 0 to 27 (28, 29). When using the PHQ-9 screening tool, a score of ten or higher is recommended as a cut-off for identifying patients with likely MDD (28). The Cronbach’s alphas for PHQ-9 are 0.851, indicating good internal consistency. Moderate to strong correlations were also seen with WHO-5, HADS-depression, HADS-anxiety, and GAD-7 measures, confirming convergent validity. Using a cut-off of ≥10, the PHQ-9 demonstrated good sensitivity (.88) and specificity (.88) (28–30).

#### 2.4.3 The World Health Organisation-Five Well-Being Index (WHO-5)

The WHO-5 is a self-reported measure of mental well-being developed by the World Health Organization. It contains five positively worded items: “I have felt cheerful and in good spirits;” “I have felt calm and relaxed;” “I have felt active and vigorous;” “I woke up feeling fresh and rested;” and “My daily life has been filled with things that interest me” (31). The frequency with which positive feelings were present in the last two weeks is assessed using a 6-point Likert scale ranging from 0 (not present) to 5 (constantly present). Raw scores are converted from 0 (worst thinkable well-being) to 100 (best thinkable well-being). Having a score <50 indicates poor emotional well-being and requires further evaluation (31). The WHO-5 demonstrated satisfactory internal consistency reliability (α = 0.90) and convergent validity with the PHQ-9 (r = −0.73, p < 0.001). It has been shown in several studies to have 93% sensitivity and 83% specificity for detecting depression (31, 32).

### 2.5 Statistical analysis

SPSS for Mac, version 25 [IBM et al., USA (33)] was used to analyze the data for this study. Using the Chi-squared test, we presented the baseline characteristics (sociodemographic data, primary and secondary diagnosis data, and clinical measures) against the gender groups of participants who completed their baseline survey before discharge. We will run the reliability test for scales under study to examine the internal consistency using Cronbach’s alpha. We used multivariate regression analysis to identify potential predictors of the three mental health conditions of the study. We included each model’s demographic and clinical factors to predict the outcome variables. Before running logistic regression, correlational analysis was performed to determine any strong intercorrelations (Spearman’s correlation coefficient of 0.7 to 1.0 or – 0.7 to – 1.0) among predictor variables). Each case was checked to ensure no violations of linearity, variance, normality, homogeneity of regression slopes, or measurement of reliable covariates had occurred. As there was no imputation for missing data, the total numbers reported represent the responses recorded for each variable. Odds ratios and confidence intervals were reported. Aiming to determine the association between each variable in the model and the likelihood of respondents presenting with anxiety, depression, and poor well-being, controlling for the other variables. The significance level was set for each analysis at two-tailed *p* < 0.05.

### 2.6 Sample size calculation

With 28,571 adult discharges from psychiatric units in Alberta in 2018, a 95% confidence interval, and a ±3% margin of error, the sample size needed for prevalence rate estimates for likely MDD, likely GAD and poor quality of life was 1,029 using an online script (34).

## 3 Result

### 3.1 Descriptive analysis

**Figure 1:**
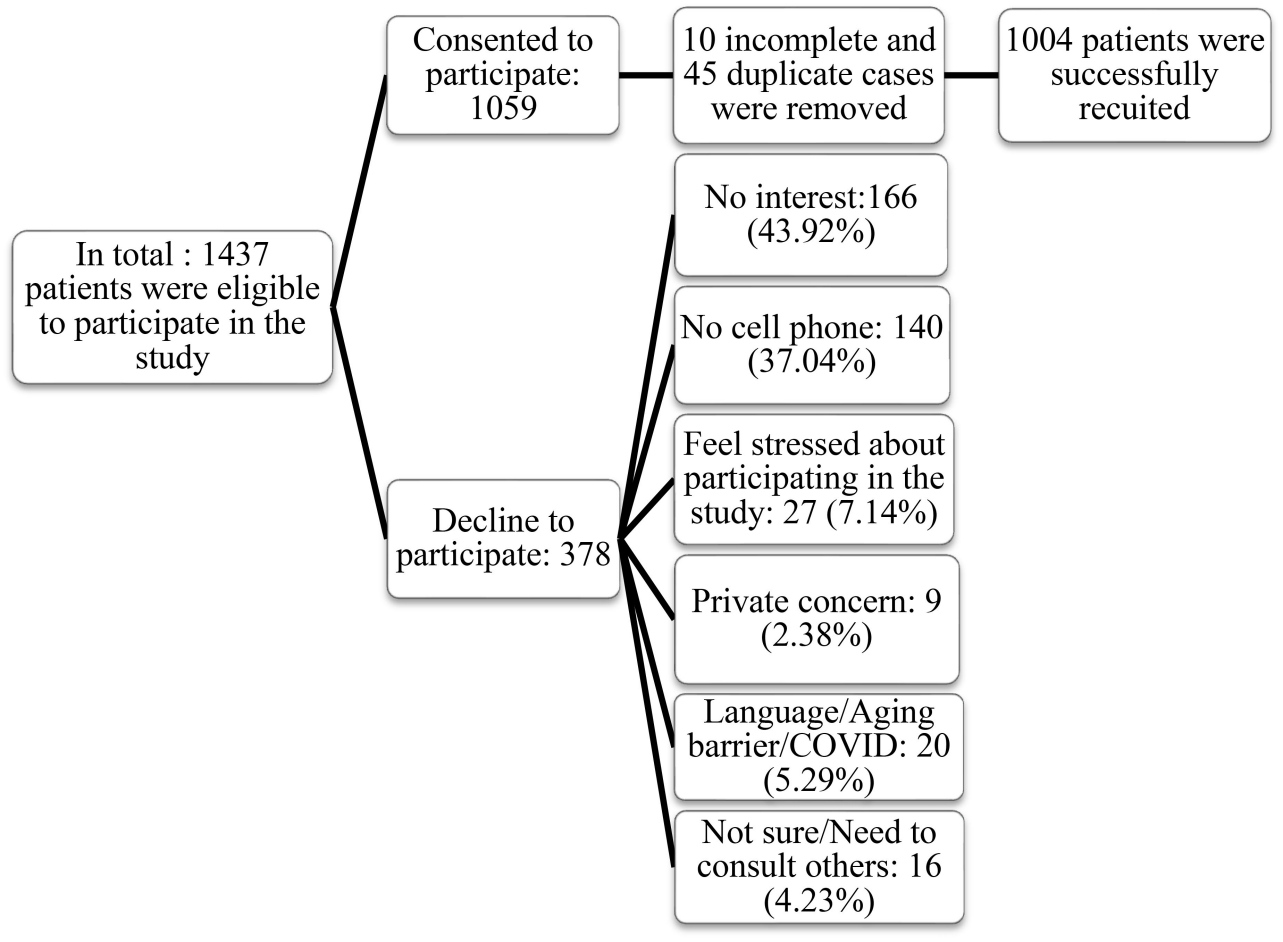
The flow chart of the recruitment process.

Overall, there were 1,059 responses, out of which 1,004 responses remained after removing five grossly incomplete cases (no socio-demographic information or response to scales), five partially incomplete cases (incomplete responses to demographic questions or scales), and forty-five duplicate cases (**Figure 1**). Demographic and clinical data were collected from the entire sample of participants (N=1,004), with gender as a comparator variable (**Table 1**). The sample included 550 (54.8%) females; 360 (35.9%) of respondents were younger than 25 years old; 296 (29.5%) were older than 40 years old; 516 (51.4%) had a high school diploma. Most respondents, 625 (62.3%) were Caucasian; 536 (53.4%) were unemployed; 591 (58.9%) were single, 412 (41.0%) lived with family or friends and 262 (26.1%) of the respondents had been diagnosed with depression. Regarding the prevalence of clinical conditions under study, a total of 370 (37.1%) of our respondents met the criteria for likely GAD, 566 (56.4%) met the criteria for likely MDD, and 484 (48.3%) met the criteria for poor well-being. **Table 1** provides a more detailed description of the characteristics of the respondents.

**Table 1:** Distribution of socio-demographic and clinical characteristics among the study participants.

Variables	Gender			
	Male (N=426) n (%) = 42.4%	Female (N=550) n (%) = 54.8%	Other gender (N=28) n (%) = 2.8%	Total (N=1,004)
Age				
≤25y 26–40y >40y	141 (33) 156 (37) 129 (30)	202 (37) 181 (33) 167 (30)	17 (61) 11 (39) 0 (0)	360 (36) 348 (35) 296 (30)
Ethnicity				
Caucasian Indigenous Black people Asian Mixed/Other	261 (61) 31 (7) 47 (11) 48 (11) 39 (9)	345 (63) 60 (11) 55 (10) 61 (11) 29 (5)	19 (68) 4 (14) 1 (4) 2 (7) 2 (7)	625 (62) 95 (10) 103 (10) 111 (11) 70 (7)
Education level				
Less than High School High School Diploma Post-secondary Education Prefer not to say	19 (5) 237 (56) 159 (37) 11 (3)	16 (3) 265 (48) 248 (45) 21 (4)	4 (14) 14 (50) 10 (36) 0 (0)	39 (4) 516 (51) 417 (42) 32 (3)
Relationship status				
Single Separated or Divorced Married/Partnered/Common-Law Widowed Prefer not to say	278 (65) 41 (10) 93 (22) 4 (1) 10 (2)	294 (54) 38 (7) 193 (35) 6 (1) 19 (4)	19 (68) 1 (4) 6 (21) 0 (0) 2 (7)	591 (59) 80 (8) 292 (29) 10 (1) 31 (3)
Employment status				
Student Employed Unemployed Retired Other	24 (6) 120 (28) 239 (56) 31 (7) 12 (3)	51 (9) 167 (30) 284 (52) 27 (5) 21 (4)	2 (7) 11 (39) 13 (46) 0 (0) 2 (7)	77 (8) 298 (30) 536 (53) 58 (6) 35 (4)
Housing status				
Own home Rented Accommodation Live with Family or Friend Couch surfing/Shelter/Street/Other	83 (20) 125 (29) 180 (42) 38 (9)	122 (22) 185 (34) 219 (40) 24 (4)	0 (0) 14 (50) 13 (46) 1 (4)	205 (20) 324 (32) 412 (41) 63 (6)
Primary Mental Health Diagnosis				
Depression Bipolar Disorder Anxiety Schizophrenia Personality Disorder Substance Use Disorder Other	95 (22) 79 (19) 50 (12) 101 (24) 18 (4) 30 (7) 53 (12)	162 (30) 123 (22) 80 (15) 56 (10) 67 (12) 20 (4) 42 (8)	5 (18) 4 (14) 5 (18) 4 (14) 6 (21) 1 (4) 3 (11)	262 (26) 206 (20) 135 (13) 161 (16) 91 (9) 51 (5) 98 (10)
GAD-7				
Unlikely Anxiety Likely Anxiety	294 (69) 131 (31)	322 (59) 221 (41)	10 (36) 18 (64)	626 (63) 370 (37)
PHQ-9				
Unlikely Depression Likely Depression	222 (52) 204 (48)	211 (38) 338 (62)	4 (14) 24 (86)	437 (44) 566 (56)
WHO-5				
Good Well-being Poor Well-being	253 (59) 173 (41)	259 (47) 290 (53)	7 (25) 21 (75)	519 (52) 484 (48)

Our reliability analysis using Cronbach’s alpha showed that there was a high internal consistency of set of items for GAD-7 (.92), PHQ-9 (.89) and WHO-5 (.90) scales among our respondents.

### 3.2 Logistic regression analysis results

#### 3.2.1 PHQ-9

The complete model was statistically significant, *χ* ^2^ (30) = (418.77, *p* <.001), indicating that it could distinguish between respondents with mild depression from those with moderate to severe depression. The model accounted for 34.3% (Cox and Snell R2) to 46.0% (Nagelkerke R2) of the variance in the likelihood that respondents will present with symptoms of MDD and accurately identified 77.8% of cases.

As shown in **Table 2**, ethnicity, primary mental health diagnoses, anxiety, and well-being at discharge independently predicted depression among respondents. Caucasians were almost two times more likely to present with symptoms of MDD prior to discharge from inpatient psychiatric unit compared to Indigenous (OR = .53; 95% CI:.30-.94) and Black people (OR = .50; 95% CI:.29-.88). Additionally, the risk of presenting with symptoms of MDD was more than twice as high in Caucasians compared with mixed or other ethnic groups (OR = .49; 95% CI:.26-.93). While housing conditions did not contribute significantly to the model, people who lived in couch surfing, shelters, on the street, or in other situations were almost three times more likely to present with symptoms of MDD than those who owned their own home (OR = 2.96; 95% CI: 1.29–6.78). The presence of the primary mental health diagnoses is another significant factor in predicting symptoms of MDD. The likelihood of presenting with symptoms of MDD prior to discharge was about two times higher for those diagnosed by the treating psychiatrist with a depressive disorder compared with those diagnosed with an anxiety disorder (OR = .58; 95% CI:.34–1.00), schizophrenia (OR = .53; 95% CI:.31-.89), bipolar diagnosis (OR = .43; 95% CI:.27-.70) and other diagnoses (OR = .53; 95% CI:.29-.98). Participants who met the criteria for likely GAD at hospital discharge had almost nine times the likelihood of developing symptoms of MDD compared to those with mild anxiety (OR = 8.84; 95% CI: 5.97–13.11). Similarly, those who met the criteria for poor well-being at discharge were four times more likely to experience MDD symptoms than those with good well-being (OR = 4.16; 95% CI: 3.00 – 5.78).

**Table 2:** Logistic regression predicting the likelihood of residents presenting with depression.

		B	S.E.	Wald	df	Sig.	Odd’s ratio	95% C.I.for EXP(B) Lower Upper	
**Age**	≤25y			1.132	2	.568			
	26–40y	-.225	.224	1.008	1	.315	.799	.515	1.238
	>40y	-.242	.275	.772	1	.380	.785	.458	1.346
**Gender**	Male			5.117	2	.077			
	Female	.324	.172	3.557	1	.059	1.383	.987	1.937
	Other	.958	.643	2.220	1	.136	2.606	.139	9.183
**Ethnicity**	Caucasian			14.150	4	.007*			
	Indigenous	-.640	.295	4.703	1	.030*	.528	.296	.940
	Black people	-.685	.286	5.756	1	.016*	.504	.288	.822
	Asian	.198	.269	.541	1	.462	1.219	.719	2.065
	Mixed/Other	-.718	.329	4.756	1	.029*	.488	.256	.930
**Education level**	Less than High School			4.561	3	.207			
	High School	-.553	.436	1.606	1	.205	.575	.245	1.353
	Post-secondary Education	-.496	.444	1.248	1	.264	.069	.255	1.454
	Prefer not to say	.302	.619	.239	1	.625	1.353	.402	4.553
**Housing status**	Own Home			7.456	3	.059			
	Rented Accommodation	.273	.253	1.177	1	.278	1.314	.802	2.152
	Live with Family or Friends	.137	.276	.247	1	.619	1.147	.688	1.969
	Coach surfing/Shelter/Street/Other	1.085	.422	6.605	1	.010*	2.961	1.294	6.776
**Primary Mental Health Diagnosis**	Depression			19.354	6	.004*			
	Bipolar	-.841	.244	11.887	1	.001*	.431	.268	.696
	Anxiety	-.542	.276	3.873	1	.049*	.581	.339	.998
	Schizophrenia	-.643	.267	5.779	1	.016*	.526	.311	.888
	Personality	.241	.348	.480	1	.488	1.273	.644	2.516
	Substance use	-.675	.389	3.019	1	.082	.509	.283	1.090
	Other	-.628	.311	4.090	1	.043*	.534	.290	.981
**GAD-7 at baseline**	Moderate to severe anxiety	2.180	.200	118.270	1	.000*	8.843	5.973	13.106
**WHO-5 at baseline**	Poor well-being	1.426	.167	72.574	1	.000*	4.164	2.999	5.782
**Constant**		-.366	.610	.360	1	.549	.694		

C.I., confidence interval. S.E., standard error. Df, degree of freedom. *Significant predictor.

#### 3.2.2 GAD-7

The logistic model was statistically significant, *χ* ^2^ (30) = (355.12, *p* <.001), showing that the model could distinguish between respondents who had moderate to severe anxiety and those at most mild anxiety. The model explained between 30.0% (Cox and Snell R^2^) and 40.9% (Nagelkerke R^2^) of the variance, in the likelihood that respondents will present with moderate to severe anxiety and correctly classified 76.8% of cases.

The results of **Table 3** show that ethnicity, education level, housing status, likely depression and poor well-being at baseline significantly predicted moderate to severe anxiety symptoms. While age did not contribute significantly to the model, respondents who were younger than 25 years old were almost two times more likely to experience GAD symptoms at hospital discharge than those who were 40 years and older (OR = .56; 95% CI:.33 -.96). Compared to Caucasians, Indigenous people were more than twice as likely to experience anxiety symptoms (OR = 2.54; 95% CI: 1.45–4.46). Respondents with a post-secondary education background were twice as likely to experience moderate to severe anxiety symptoms compared with those with less than high school education (OR = 2.43; 95% CI: 1.07–5.53). People who rented accommodations were almost twice as likely to experience moderate to severe anxiety as those who owned their own homes (OR = 1.80; 95% CI: 1.10–3.00) when assessing housing status. Furthermore, participants who reported poor well-being were three times more likely to experience moderate to severe anxiety than those with good well-being at discharge (OR = 2.80; 95% CI: 2.01–4.00); and similarly, those who met criteria of moderate to severe depression were more than eight times more likely to experience moderate to severe anxiety symptoms than those with mild depression (OR = 8.50; 95% CI: 5.80–12.60).

**Table 3:** Logistic regression predicting the likelihood of residents presenting with anxiety.

		B	S.E.	Wald	df	Sig.	Odd’s ratio	95% C.I.for EXP(B) Lower Upper	
**Age**	≤25y			5.377	2	.068			
	26–40y	-.100	.217	.212	1	.645	.905	.591	1.385
	>40y	-.575	.271	4.515	1	.034*	.563	.331	.956
**Gender**	Male			.873	2	.646			
	Female	.114	.174	.428	1	.513	1.121	.797	1.576
	Other	.388	.490	.625	1	.429	1.474	.564	.3.853
**Ethnicity**	Caucasian			12.590	4	.013*			
	Indigenous	.935	.286	10.672	1	.001*	2.547	1.453	4.462
	Black people	-.082	.294	.077	1	.781	.921	.517	1.641
	Asian	-.192	.265	.528	1	.467	.825	.491	1.386
	Mixed/Other	.143	.335	.183	1	.669	1.154	.599	2.225
**Education level**	Less than High School			10.279	3	.016*			
	High School	.469	.411	1.304	1	.253	1.599	.715	3.577
	Post-secondary Education	.889	.419	4.509	1	.034*	2.433	1.071	5.530
	Prefer not to say	-.271	.637	.182	1	.670	.762	.219	2.655
**Housing status**	Own Home			9.667	3	.022*			
	Rented Accommodation	.591	.252	5.516	1	.019*	1.807	1.103	2.960
	Live with Family or Friends	.134	.282	.227	1	.634	1.144	.658	1.988
	Coach surfing/Shelter/Street/Other	.720	.388	3.439	1	.064	2.055	.960	4.398
**Primary Mental Health Diagnosis**	Depression			3.102	6	.796			
	Bipolar	-.212	.243	.761	1	.383	.809	.502	1.302
	Anxiety	.063	.269	.056	1	.814	1.066	.629	1.806
	Schizophrenia	.014	.267	.003	1	.958	1.014	.601	1.711
	Personality	-.373	.302	1.526	1	.217	.689	.381	1.245
	Substance use	.185	.404	.210	1	.647	1.203	.575	2.657
	Other	-.132	.318	.171	1	.679	.877	.470	1.636
**PHQ-9 at baseline**	Moderate to severe depression	2.140	.199	115.760	1	.000*	8.500	5.756	3.957
**WHO-5 at baseline**	Poor well-being	1.038	.172	36.289	1	.000*	2.823	2.014	3.957
**Constant**		-3.044	.595	26.132	1	.000	.048		

C.I., Confidence interval; S.E., Standard error; Df, Degree of freedom; *Significant predictor.

#### 3.2.3 WHO-5

The model predicting poor well-being was statistically significant, *χ* ^2^ (30) = (270.31, *p* <.001), demonstrating that the model could distinguish between respondents who had good well-being and poor well-being. The model explained between 23.8% (Cox and Snell R2) and 31.7% (Nagelkerke R2) of the variance in the likelihood that respondents will present with poor well-being and accurately classifies 72.5% of the case.

**Table 4** illustrates that only two independent variables, presence of moderate to severe depression and anxiety symptoms contributed significantly to the model for predicting respondents’ well-being. Although primary mental health diagnosis did not contribute significantly to the model, at hospital discharge, people who had a depression diagnosis were two times more likely to present with poor well-being symptoms compared with those with a primary substance use disorder diagnosis (OR = .45; 95% CI:.23-.99). Respondents who met criteria for likely GAD were more than four times more likely (OR = 4.07; 95% CI: 2.93–5.64) to present with poor well-being than those experiencing at most mild depression. Similarly, respondents who met the criteria for likely MDD were almost three times more likely to present with poor well-being than respondents with the mildest anxiety (OR = 2.85; 95% CI: 2.04–3.99).

**Table 4:** Logistic regression predicting the likelihood of residents presenting with well-being.

		B	S.E.	Wald	df	Sig.	Odd’s ratio	95% C.I. for EXP(B) Lower Upper	
**Age**	<=25y			4.180	2	.124			
	26–40y	-.253	.201	1.578	1	.209	.777	.524	1.152
	>40y	.147	.248	.349	1	.555	1.158	.712	1.883
**Gender**	Male			3.853	2	.146			
	Female	.213	.158	1.819	1	.177	1.238	.908	1.688
	Other	.825	.502	2.704	1	.100	2.282	.854	6.099
**Ethnicity**	Caucasian			5.258	4	.262			
	Indigenous	-.423	.268	2.495	1	.114	.655	.387	1.107
	Black people	-.131	.259	.255	1	.614	.878	.529	1.457
	Asian	-.380	.246	2.373	1	.123	.684	.422	1.109
	Mixed/Other	-.389	.307	1.605	1	.205	.677	.371	1.237
**Education level**	Less than High School			1.387	3	.709			
	High School	-.302	.394	.587	1	.444	.739	.341	1.602
	Post-secondary Education	-.427	.401	1.134	1	.287	.653	.297	1.432
	Prefer not to say	-.405	.558	.528	1	.467	.667	.223	1.990
**Housing status**	Own Home			1.896	3	.594			
	Rented Accommodation	-.252	.230	1.206	1	.272	.777	.495	1.219
	Live with Family or Friends	-.271	.253	1.153	1	.283	.762	.465	1.251
	Coach surfing/Shelter/Street/Other	-.439	.362	1.468	1	.226	.645	.317	1.311
**Primary Mental Health Diagnosis**	Depression			9.617	6	.142			
	Bipolar	-.319	.221	2.097	1	.148	.727	.472	1.120
	Anxiety	-.232	.250	.861	1	.353	.793	.485	1.295
	Schizophrenia	-.335	.243	1.892	1	.169	.716	.444	1.153
	Personality	.313	.297	1.114	1	.291	1.368	.765	2.446
	Substance use	-.741	.374	3.913	1	.048*	.477	.229	.993
	Other	.013	.283	.002	4	.964	1.013	.581	1.765
**GAD-7 at baseline**	Moderate to severe anxiety	1.403	.167	70.768	1	.000*	4.069	2.934	5.643
**PHQ-9 at baseline**	Moderate to severe depression	1.048	.171	37.583	1	.000*	2.851	2.039	3.985
**Constant**		-.655	.551	1.416	1	.234	.519		

C.I., Confidence interval; S.E., Standard error; Df, Degree of freedom; *Significant predictor.

## 4 Discussion

This study aimed to determine the prevalence and predictors of several mental health conditions among patients at their time of discharge from psychiatric units at ten primary sites throughout the province of Alberta. Among our respondents, anxiety, depression, and poor well-being were reported at 37.1%, 56.4%, and 48.3%, respectively, indicating that around half of the patients were experiencing a certain degree of mental health concern at the time of discharge. Our findings demonstrated that at discharge, the rates of mental health problems remained much greater than the self-reported prevalence of depression (11.3%), anxiety (14%), and poor well-being (38.1%) among the general psychiatric outpatient population based on 2012 Canadian Community Health Survey (CCHS) on Mental Health (35). Aside from the possibility that some patients’ conditions did not effectively respond to treatment, another explanation is that in most cases, hospitalization is necessary due to the severity of the individual’s condition, which cannot be effectively or safely managed in an outpatient psychiatric setting. This study did not measure or gather data related to the mental status of participants on admission. However, given that the multidisciplinary inpatient treatment teams deemed each of these patients had improved to the extent that they could continue with their treatment in the community, it is highly probable, that their mental health status on admission was far worse than their discharge mental health status. Thus, it is likely that patients had far worse depressive, anxiety, and poor well-being symptoms on admission, and so the prevalence and severity of these conditions at the time of discharge, although high, still represent an improvement from what they might have been on admission. Also, patients suffering from conditions such as persistent chronic depression or borderline personality disorder may continue to have depression or suicidal thoughts even until discharge, which does not mean they are necessary to be kept in the hospital all the time (36, 37). Additionally, the high level of depression, anxiety, and poor well-being may also be related to the self-reported scales we used, which may not clinically reflect mental health conditions. In addition, part of the nature of this study is that participants were recruited from acute care units that were primarily focused on stabilization and linking them with supportive services in the community. It is not surprising to observe some patients who were about to be discharged have high levels of depression, anxiety, or poor well-being as they continue their recovery in the community.

Although we did not find any research regarding mental health status at discharge among patients from the Psychiatry unit, our study results are comparable to those of some other studies on the same research topic among patients from a variety of different hospital departments. In a study that investigated anxiety and depression among medical and surgical patients who were close to being discharged from the hospital, 36% of patients were found to be clinically depressed or anxious (38). In another study conducted on 42 individuals about to return home after inpatient neurorehabilitation treatment, it was determined that nearly half of the participants (n = 19; 45%) experienced clinically significant levels of anxiety before discharge (20). As evidenced by the limited but alarming rates shown in this research, the mental health status of patients at the time of discharge from not only psychiatry departments but from all medical divisions is highly relevant. A thorough investigation and application of effective interventions and policies is necessary.

Previous research has demonstrated that depression and anxiety symptoms are highly correlated (24, 39). A study by Clayton found that nearly two-thirds of 327 patients with depression also had anxiety symptoms (40). In terms of anxiety disorders, the lifetime comorbidity with depression is estimated to range from 20% to 70% (21). There are some possible explanations for why depression and anxiety are often paired together. A theory suggests that similar biological mechanisms in the brain cause the two conditions, so they are more likely to show up together (41). Another explanation is that both diagnoses have many symptoms that overlap, making it common for people to meet both criteria (41). As a result, it is not surprising to see that patients with high levels of depression are also prone to developing moderate to severe anxiety symptoms and vice versa, especially during stressful or critical life moments such as hospital discharge. Further, the inverse relationship between depression, anxiety, and well-being in our study was also examined in a mainland Chinese sample (24). The results suggested that anxiety and depression were inversely related to measures of well-being. That is, the higher the prevalence of depression and anxiety, the more likelihood of poor well-being. Similar relationships have been found in the United States (42, 43) Germany, Japan, and South Korea (44).

In predicting likely depression at discharge, it is not surprising that people with pre-existing “other diagnoses” were less likely than those with pre-existing depression diagnosis to exhibit depression symptoms at the time of discharge. The results were similar for predicting well-being at discharge, with the only significant variable being that people with substance use disorders were less likely to experience poor well-being than those with depression diagnoses. By WHO’s explanation of depression, this outcome may be explained by the fact that depression is a mental health condition that can negatively impact physical health as well (45). It may affect a patient’s cardiovascular system, digestive system, and immune system. A person suffering from depression may experience disturbed sleep and changes in appetite. It is also common for them to feel tired and unable to concentrate almost all the time. They may have feelings of low self-worth, thoughts of dying, and feelings of hopelessness about the future. As a result, it adversely affects patients’ quality of life and well-being (45). Considering the high co-occurrence of depression and substance abuse disorders, future research in the field would be of interest if it included patients with co-morbidities of both conditions.

This study revealed interesting results regarding ethnicity. People of Caucasian ethnicity was more likely than those from other ethnicities to experience MDD symptoms at discharge; while Indigenous people were more likely to experience anxiety. The results regarding depression were controversial. There is existing research that indicates that marginalized racial and ethnic groups, such as black people and Hispanics, were more prone to experiencing severe and debilitating mental health symptoms, which could be due to a variety of factors, such as cost or lack of adequate health insurance coverage (46–49). Finding providers of one’s race or ethnicity may also be difficult (49). People may also be prevented from seeking mental health care due to stigma or negative perceptions of mental healthcare (46–49). On the other hand, the findings of our study were also supported by studies that showed that minority populations suffer from acute depression at a lower rate than Caucasians (50). It has been argued that African Americans may have lower rates of depression than Caucasians due to the resilience of their communities and their greater religious support. However, some may argue that these patients are usually underdiagnosed or misdiagnosed (51).

Housing status is another shared variable that was closely associated with MDD and GAD symptoms at discharge; individuals who reported living in places such as couch surfing, shelters, streets, or rented accommodations were found to be at a higher risk of depression and anxiety than those who own their own homes. Understandably, housing status is perceived as one of the main health determinants that has a causal relationship with mental health. Studies have shown that people with less severe mental health challenges can suffer from aggravated symptoms as a result of a lack of stable housing. Thus access to more stable housing can assist in alleviating some of these symptoms (52) and should be considered as part of discharge planning for patients admitted to psychiatric units. In a recent study, stable housing was found to reduce anxiety and improve variables influencing mental health, including anxiety and stress (53). During the period before discharge, when the patient has no idea where he or she will live after leaving the hospital, or what they may expect is a hard environment and poor living conditions, it is reasonable for the patient to experience depression or anxiety symptoms.

Respondents’ age and educational background also predicted GAD symptoms at the time of hospital discharge. GAD symptoms were less likely to occur among patients over 40 years of age as compared to those who were under 25 years of age, and participants with post-secondary education were more likely to exhibit anxiety than those with less than high school educational backgrounds. In terms of age, it has been reported in several publications that the elderly were less vulnerable to the psychological impacts of stressors than younger people (54–56). Two theories were proposed in linking old age with mental illness. “Maturation theory” suggests that older adults possess more mature coping mechanisms that protect them against stressors (57). Therefore, elderly people tend to respond less negatively to stressful life events. Inoculation theory believes the result may be due to the knowledge and experience of older individuals in developing coping skills in front of difficulties (57). However, according to the “resource theory”, elderly individuals are more prone to psychological problems and are unable to fully recover due to their lower socioeconomic status and limited functional abilities (58, 59).

Regarding the hospital discharge setting, one possible explanation for our finding is that individuals with higher education levels are more likely to have relatively higher job-related stress (60), the work suspended due to hospitalization may cause more psychological stressors, especially at the time before discharge. Further, individuals with higher educational backgrounds are more likely to have high socioeconomic status (SES). Hospitalization due to mental health issues may cause feelings of self-doubt, and have a stronger effect on self-evaluation and self-esteem for those with higher socioeconomic status, who are generally more in control and have a higher expectation of their lives In particular, mental uncertainty can cause significant mental health problems when people have to face their regular daily routine again without knowing if they can manage it well this time.

At present, there is limited and inconsistent evidence regarding education’s effects on mental health problems such as anxiety. Contrary to our findings, much of the available literature holds that a high education background is protective against mental health issues such as anxiety as education tends to lead to greater wealth, more choice, and, as a result, a greater sense of control over one’s life and increased security (61). A systematic review examined the relationship between anxiety and social inequalities found that education level had a significant negative association with anxiety disorders (62). Another longitudinal study on a similar topic demonstrated that respondents’ anxiety level in front of stressors was negatively related to both parental education and their level of education (63). Despite this, some studies are consistent with our findings (64–66). A study published in the Lancet Public Health indicated that highly educated young people have higher levels of anxiety than their peers in front of stressors (66). There have been several studies conducted in the UK that have found no protective effects associated with education (67), and Avendano has even found a piece of evidence that more education results in worse mental health (68).

## 5 Limitations

Our study is not without limitations. For example, with a sample size of 1,004 rather than the 1,029 we had expected, our estimations of the prevalence rates for anxiety, depression, and poor quality of life have a margin of error of ±3.09% at 95% confidence intervals rather than the ±3% we had anticipated. Also, this study relies on self-reported measuring scales, such as GAD, PHQ, and WHO-5 Well-being Index, which may lead to bias and adversely impact the objectivity of the information provided by respondents. A further limitation is, details regarding patients’ treatment and hospital services were not measured, such as treatment method, treatment duration and their mental status on admission, which could potentially influence participants’ mental health status upon discharge. While assessment of these factors was not the goal of this epidemiological study, the lack of such information necessarily limits our conclusions. Nonetheless, while there are limitations to this study as described above, it contributes to the current limited literature that is available in the mental health field concerning discharge and recovery in Canada.

## 6 Conclusion

This study investigated the prevalence and potential risk factors associated with depression, anxiety, and poor well-being symptoms among Albertan patients who were about to be discharged from psychiatric units. Among all the participants, 31.7% met the criteria for likely MDD, 56.4% met the criteria for likely GAD, and 48.4% met the criteria for poor well-being. In addition, ethnicity, education level, housing status, primary mental health diagnoses, and baseline mental health status are significantly associated with the mental health conditions of respondents who are close to discharge.

Patients’ mental health status at each stage of their treatment must be given due attention to maximize the benefits of inpatient psychiatry treatment, promote recovery, and prevent relapses and readmissions. Before discharge, doctors should address patients who still exhibit high levels of symptoms. An extended hospital stay may be considered, and close follow-up and effective supportive interventions following discharge are also necessary. There should be widespread application of supportive resources and interventions in the community, such as supportive text messaging and peer support programs (69, 70), to facilitate patient recovery and enhance quality of life following hospital discharge. Further research is required to better understand the progression and prognosis of mental health status among patients with various psychiatric disorders. As a result of a greater understanding of recovery dynamics, healthcare providers will be able to tailor interventions and support systems to better address the diverse needs of patients discharged from acute psychiatric care. Future research may help clarify these factors more, and ideally will be able to augment these findings by studying patients early during their admission as well as immediately prior to discharge, and collect appropriate information about the treatment received by each patient.

## Data availability statement

The original contributions presented in the study are included in the article/supplementary material. Further inquiries can be directed to the corresponding author.

## Ethics statement

Ethical approval for this study was obtained from the University of Alberta’s Health Research Ethics Board (Ref # Pro00111459). The studies were conducted in accordance with the local legislation and institutional requirements. The participants provided their written informed consent to participate in this study. Written informed consent was obtained from the individual(s) for the publication of any potentially identifiable images or data included in this article.

## Author contributions

VA: Conceptualization, Data curation, Formal analysis, Funding acquisition, Investigation, Methodology, Project administration, Resources, Supervision, Writing – review & editing. WM: Data curation, Formal analysis, Methodology, Writing – original draft. RS: Data curation, Formal analysis, Methodology, Writing – review & editing. EO: Data curation, Formal analysis, Methodology, Writing – review & editing. HE: Data curation, Formal analysis, Methodology, Writing – review & editing. BA: Data curation, Formal analysis, Methodology, Writing – review & editing. EE: Data curation, Formal analysis, Methodology, Writing – review & editing. PS: Methodology, Supervision, Writing – review & editing. PC: Methodology, Supervision, Writing – review & editing. X-ML: Methodology, Supervision, Writing – review & editing. WV: Methodology, Supervision, Writing – review & editing. AO: Methodology, Supervision, Writing – review & editing. VT: Methodology, Supervision, Writing – review & editing. AG: Methodology, Supervision, Writing – review & editing.

## Appendix I

**Table:** Text4Support plus peer support project team (T4SPPSP-Team).

Last Name	First Name	Primary Organization	Organizational Position
Agyapong	Vincent	Department of Psychiatry, Dalhousie University	Professor and Head
Bales	Kerry	Addiction and Mental Health Strategic Clinical Network	Senior Program Officer
Taylor	Valerie	Department of Psychiatry, University of Calgary	Head
Greenshaw	Andrew	Department of Psychiatry, Faculty of Medicine and Dentistry, University of Alberta	Associate Chair
Ohinmaa	Arto	School of Public Health, University of Alberta and Institute of Health Economics	Professor
Hilario	Carla	Faculty of Nursing, University of Alberta	Assistant Professor
Greiner	Russ	Faculty of Science, University of Alberta	Professor
Silverstone	Peter	Department of Psychiatry, University of Alberta & Edmonton Zone Community Mental Health	Professor
McCabe	Christophe	Institute for Health Economics	Executive Director/CEO
Bremault Phillips	Suzette	Heroes in Mind, Advocacy and Research Consortium (HiMARC)/University of Alberta	Director HiMARC and Associate Professor
Cao	Bo	Department of Psychiatry, Faculty of Medicine and Dentistry, University of Alberta	Assistant Professor
MacMaster	Frank	Addiction and Mental Health Strategic Clinical Network	Scientific Director
Bales	Kerry	Addiction and Mental Health Strategic Clinical Network	Senior Program Officer
Snaterse	Mark	Addiction and Mental Health, Alberta Health Services, Edmonton Zone	Executive Director
Chafe	Janet	Addiction and Mental Health, Alberta Health Services, Calgary Zone	Executive Director
Greening	Stacy	QEII Regional Hospital and North Zone, Addictions and Mental Health Alberta Health Services	Senior Operating Officer
Given	Susan	Addiction and Mental Health, Alberta Health Services, North Zone	Executive Director
McLean	Carla	Addiction and Mental Health, Alberta Health Services, North East Zone	Director
Rittenbach	Katherine	Addiction and Mental Health Strategic Clinical Network	Assistant Scientific Director
Li	Xin-Min	Department of Psychiatry, Faculty of Medicine and Dentistry, University of Alberta	Chair
Rathwell	Rebecca	Edmonton Zone Peer Support Practice Council	Co-chair
Knox	Michelle	Housing and Recovery Supports Young Adult and Cross Level Services Addiction and Mental Health, Edmonton Zone	Program manager
Chue	Pierre	Department of Psychiatry, Faculty of Medicine and Dentistry, University of Alberta	Clinical Professor
Abda-Aji	Adam	Department of Psychiatry, Faculty of Medicine and Dentistry, University of Alberta	Associate Clinical Professor
Surood	Shireen	Information, Evaluation & Professional Practice Addiction & Mental Health, Alberta Health Services	Manager
Nwachukwu	Izu	Department of Psychiatry, University of Calgary	Associate Clinical Professor
Vuong	Wesley	Decision Support Services - Business Intelligence Information, Evaluation & Professional Practice Addiction & Mental Health - Edmonton Zone	Research & Evaluation Coordinator
Shalaby	Reham	Department of Psychiatry, Faculty of Medicine and Dentistry, University of Alberta	PhD Candidate
Berhe	Tzeggai	QEII Regional Hospital and North Zone, Addictions and Mental Health Alberta Health Services	Zone Clinical Facility Chief
Ambrosano	Lorella	Northern Lights Regional Health Centre	Zone Clinical Facility Chief for Addiction and Mental Health
Grauwiler	David	Canadian Mental Health Association, Alberta	Executive Director
Jordan	Sara	Canadian Mental Health Association, Calgary Zone	Executive Director
Lindy	Fors	Patients’ Advisory Council, Addiction and Mental Health, Alberta Health Services	Patient Advisor
Brown	Ed	Patients’ Advisory Council, Addiction and Mental Health, Alberta Health Services	Patient Advisor
Savard	Tyla	Patients’ Advisory Council, Addiction and Mental Health, Alberta Health Services	Patient Advisor
Spurvey	Pamela	Addiction and Mental Health, Alberta Health Services, Edmonton Zone	Peer support worker
Grauwiler	David	Canadian Mental Health Association, Alberta Division	Executive Director
Grunau	Mara	Centre for Suicide Prevention	Executive Director
Frank	Kelton	Potential Place	Executive Director
Sheila	Stauffer	Cornerstone Counselling	Executive Director
